# IRaPPA: information retrieval based integration of biophysical models for protein assembly selection

**DOI:** 10.1093/bioinformatics/btx068

**Published:** 2017-02-14

**Authors:** Iain H Moal, Didier Barradas-Bautista, Brian Jiménez-García, Mieczyslaw Torchala, Arjan van der Velde, Thom Vreven, Zhiping Weng, Paul A Bates, Juan Fernández-Recio

**Affiliations:** 1 European Molecular Biology Laboratory, European Bioinformatics Institute, Wellcome Trust Genome Campus, Hinxton, Cambridge, UK; 2 Life Science Department, Joint BSC-IRB Research Program in Computational Biology, Barcelona Supercomputing Center, Barcelona, Spain; 3 Biomolecular Modelling Laboratory, The Francis Crick Institute, London, UK; 4 Program in Bioinformatics and Integrative Biology, University of Massachusetts Medical School, Worcester, MA, USA; 5 Bioinformatics Program, Boston University, Boston, MA, USA

## Abstract

**Motivation:**

In order to function, proteins frequently bind to one another and form 3D assemblies. Knowledge of the atomic details of these structures helps our understanding of how proteins work together, how mutations can lead to disease, and facilitates the designing of drugs which prevent or mimic the interaction.

**Results:**

Atomic modeling of protein–protein interactions requires the selection of near-native structures from a set of docked poses based on their calculable properties. By considering this as an information retrieval problem, we have adapted methods developed for Internet search ranking and electoral voting into IRaPPA, a pipeline integrating biophysical properties. The approach enhances the identification of near-native structures when applied to four docking methods, resulting in a near-native appearing in the top 10 solutions for up to 50% of complexes benchmarked, and up to 70% in the top 100.

**Availability and Implementation:**

IRaPPA has been implemented in the SwarmDock server (http://bmm.crick.ac.uk/∼SwarmDock/), pyDock server (http://life.bsc.es/pid/pydockrescoring/) and ZDOCK server (http://zdock.umassmed.edu/), with code available on request.

**Supplementary information:**

Supplementary data are available at *Bioinformatics* online.

## Introduction

Interactions between proteins are central to biology, from intracellular and intercellular signal transduction to the mesoscopic assemblies which regulate cell morphology. Although a full understanding of these interactions can only be gained through consideration of their atomic details, the rate at which structures are determined lags far behind the rate interactions are discovered, with only 7% of the known human interactome structurally characterized (Mosca *et al.*, 2013). Protein–protein docking can close this gap and potentially increase coverage to 50% (Mosca *et al.*, 2013). A major impediment, however, is the ability to pick out the correct solution from a set of decoys generated by a docking program.

The task of ranking docked poses is achieved by a function whose arguments are a set of predicted structures and output is an ordered list of the same structures. For some complexes this can be achieved successfully using functions based on physical models for which docked poses were not used in their formulation (Camacho *et al.*, 2000; Gabb *et al.*, 1997; Moal and Fernández-Recio, 2013; Moont *et al.*, 1999; Moal *et al.*, 2015b; Wodak *et al.*, 1987; Zacharias, 2003). However, with the growing availability of structural data, functions that are trained using docking decoys tend to have better success rates. These *ad-hoc* functions range from weighted terms in a linear combination of metrics such as electrostatic and desolvation energy (Cheng *et al.*, 2007; Pierce and Weng, 2007), to pair potentials calculated directly from structure (Chuang *et al.*, 2008; Tobi, 2010). The form of the scoring function, the objective function used to parameterize it, and the method used to solve the objective function vary; Tobi (2010) use linear programming to derive a potential function to satisfy the constraints that non-native decoys should be higher in energy than the native structure of the same complex. Cheng *et al.* (2007) and Pierce and Weng (2007) employ the simplex method to optimize term weights so as to minimize the sum of the ranks of the top-ranked near-native decoy, with Cheng *et al.* (2007) optimizing the logarithm of the ranks to shift the focus away from the poorly ranked complexes and towards cases for which near-native structures could be highly ranked. The common factor in these approaches is the use of domain knowledge to tailor the method to the docking problem: the implicit acknowledgement that the scores of decoys only matter relative to those of other decoys of the same complex and it need not be the case that the score of a near-native for one complex be better than a non-native decoy of a different complex; the recognition that a low-ranking near-native should not be considered detrimental to the performance of the method as long as a different near-native solution is high-ranking; and accounting for the fact that improving a near-native ranking of, for example 10 positions, is a lot more significant when going from rank 11 to rank 1 than going from rank 411 to rank 401.

The sorts of idiosyncrasies outlined above are not addressed by ’black box’ machine learning approaches. A standard binary classifier for distinguishing near-natives from non-natives may struggle with class imbalance due to the large number of non-natives relative to near-natives. Further, a hard threshold to distinguish between the two classes is coarse and ignores the fact that while non-native structures may be categorically incorrect, near-natives have a gradation of accuracy. A standard regression model against quality measures such as RMSD or DockQ score (Basu and Wallner, 2016) would be unaware that only the ordering of top-scoring decoys matters and unduly attempt to order structures far from the binding site. Both regression and classification would require the lumping together of decoys from different complexes. However, a procedure with many parallels to the ranking of docked poses is undertaken by Internet search engines. When searching for a query, the engine identifies many documents which match the query and characterizes them using metrics such as the number of times the query appears in the document. Similarly, docking a complex also undertakes a search, a conformational search, and the returned conformations are characterized by metrics such as statistical potentials. Search engines use these metrics to return a ranked list of the documents where only the first page of search hits, the topmost ranked items, are usually considered by the user, just as only the top-ranked poses are generally considered by the users of docking software. The web-search algorithms are trained from a set of queries and their corresponding document lists, partially ordered by document relevance. Consequently, the same algorithms can be used to train ranking functions from sets of complexes and their corresponding decoy sets, partially ordered by the evaluation criteria developed by the docking community over the early rounds of the CAPRI experiment (Lensink *et al.*, 2007). These algorithms have received much attention due to their ubiquity and importance, with the field being stimulated by competitions such as the Internet Mathematics contest in 2009, the Yahoo! Learning to Rank Challenge in 2010, and the ICDM Expedia Challenge in 2013. However, the protein–protein docking community has not yet exploited this equivalence and embraced the tools and conceptual frameworks used in the design of information retrieval systems (Moal *et al.*, 2013).

**Fig. 1:**
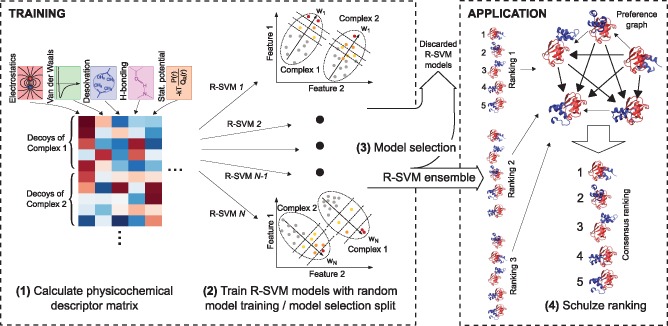
An overview of the algorithm. 1: The training decoys are characterized using physicochemical descriptors, which are organized into a matrix (see Section 2.1). 2: R-SVMs are calculated with a random model training and model selection split (Section 2.3). Each R-SVM generates a weight vector (**w**) in descriptor space such that decoys for each complex (ellipses), when projected upon it, are ordered to minimize the number of swapped pairs relative to a perfect ranking: high quality (red) > medium quality (orange) > acceptable (yellow) > incorrect (gray) (Section 2.4). 3: The highest performing models are selected according to their performance on the model selection set (Section 2.5), and form the R-SVM ensemble. 4: When applied to a new set of decoys, rankings from each R-SVM in the ensemble are combined into a graph whose edge weights indicate the number of times each pose (node) is ranked higher than each other pose. For each pair of poses, a pairwise ranking is obtained by finding the strongest directed path between them, from which the final consensus ranking follows (Section 2.6) (Color version of this figure is available at *Bioinformatics* online.)

Here, we present IRaPPA (Integrative Ranking of Protein–Protein Assemblies), a method to select and combine physicochemical descriptors for ranking docked poses (Fig. 1 and Methods). Decoys are characterized with a large selection of metrics (Moal *et al.*, 2015a), including biophysical models, statistical potentials and composite energy functions. These are combined using ranking support vector machines (R-SVMs), an efficient method that is popular for information retrieval (Joachims, 2002). As the ranking of decoys does not need to be performed instantaneously, as would be required for an Internet search engine, we take advantage of the accuracy and stability of aggregating an ensemble of learners (Breiman, 1996). To combine the individual rankings of the R-SVM ensemble, we generate a consensus ranking using the Schulze voting method, a common method that is popular due to its ease of implementation and desirable rank aggregation properties (Schulze, 2011). Our approach was applied independently to decoy structures from four state of the art docking programs, SwarmDock (Moal and Bates, 2010), pyDock (Cheng *et al.*, 2007), ZDOCK (Chen *et al.*, 2003) and SDOCK (Zhang and Lai, 2011).

## 2 Materials and methods

### 2.1 Biophysical functions

Physicochemical descriptors were calculated in an identical manner to the CCharPPI server (Moal *et al.*, 2015a). The descriptors suffixed with _MIN or _CB were omitted due to computational cost, and the pyDock and SIPPER terms were omitted due to difficulties with the computing cluster used. The NSC and NIPacking descriptors were also discarded due to a large number of failures, leaving an overall failure rate of 0.16%. The corresponding missing descriptor values were imputed as the mean within the docking method. We also included cluster sizes as descriptors, calculated with the g_cluster tool in GROMACS (Pronk *et al.*, 2013), using single-linkage clustering of ligand C*α* positions after superposition on the receptor, with cut-offs at 0.5 Å intervals in the 3–7 Å range. This gave 91 features in total, the values of which were normalized as z-scores. See Supplementary Table S2 for a complete feature list and Moal *et al.* (2013) for a more detailed description of the features.

### 2.2 Decoy sets

The interactions used in this study were taken from the docking benchmark 5.0 (Vreven *et al.*, 2015). SwarmDock decoys were obtained from the SwarmDock server (Torchala *et al.*, 2013), generating around 480 poses per complex. ZDOCK 3.0.1 was run using dense angular sampling (Chen *et al.*, 2003), pyDock was run using a grid size of 0.7 Å, surface thickness of 1.3 Å, and rotational step of $12^{{}^{\circ}}$ (Cheng *et al.*, 2007), and SDOCK poses were generated using default settings (Zhang and Lai, 2011). To reduce the computational cost of training, which scales linearly with the number of complexes but quadratically with the number of decoys within a complex, we only consider the top 500 structures from the latter three programs, and thus discard the 41, 51 and 55 complexes for SDOCK, pyDock and ZDOCK respectively, for which a near native can be found but is ranked below 500. Of these 8, 15 and 14 are from the benchmark 5.0 update. Poses were classified as incorrect, acceptable, medium quality, or high quality, using the CAPRI criteria, outlined in Lensink *et al.* (2007). For the 55 new complexes in the benchmark 5.0 update (Vreven *et al.*, 2015), the number of complexes for which a near-native could be found was 41, 33, 25 and 32, for SwarmDock, pyDock, ZDOCK and SDOCK respectively. For the 176 benchmark 4 complexes (Hwang *et al.*, 2010), these are 126, 103, 114 and 109. Affinity, flexibility and type data were obtained from the benchmark and Kastritis *et al.* (2011).

### 2.3 Overview of training, validation and metaparameter optimization

Once the decoys were classified according to the CAPRI criteria and characterized using the descriptors, the process for training and validating the models consisted of several steps. These steps were performed independently for the four docking methods, using the decoys generated by that docking method. Additionally, two different procedures were taken for validating the method (denoted *A* and *B*), such that the steps below were repeated eight times.

1. The complexes were split into multiple sets:
  - *A:* The BM4 complexes were randomly partitioned into a training set and model selection set with a 2:1 ratio, and the new complexes in the BM5 set form the test set. This was repeated 200 times.
  - *B:* All complexes were randomly partitioned into training, model selection and test set in a 2:1:1 ratio. This was repeated 800 times, so that for each complex there are approximately 200 splits for which that complex is in the test set.
2. The training sets were used to train ranking support vector machines (R-SVMs, see Section 2.4).
3. Each R-SVM was scored by applying it to its corresponding model selection set (see Section 2.5).
4. Model selection and application to test set:
  - *A:* All but the top scoring *n* R-SVM models are discarded. These are applied to the BM5 test set to produce *n* rankings for each complex.
  - *B:* For each complex in the BM4, all but the top scoring *n* R-SVM models for which that complex is in its corresponding test set, and thus not used for training or model selection, are discarded. The remaining R-SVM models are applied to that complex. Thus, *n* rankings are produced for each complex.
5. The rankings for each complex are combined using the Schulze method (Section 2.6) to produce a consensus ranking for each complex. These rankings are used to calculate the retrieval rates and success rates.

**Fig. 2:**
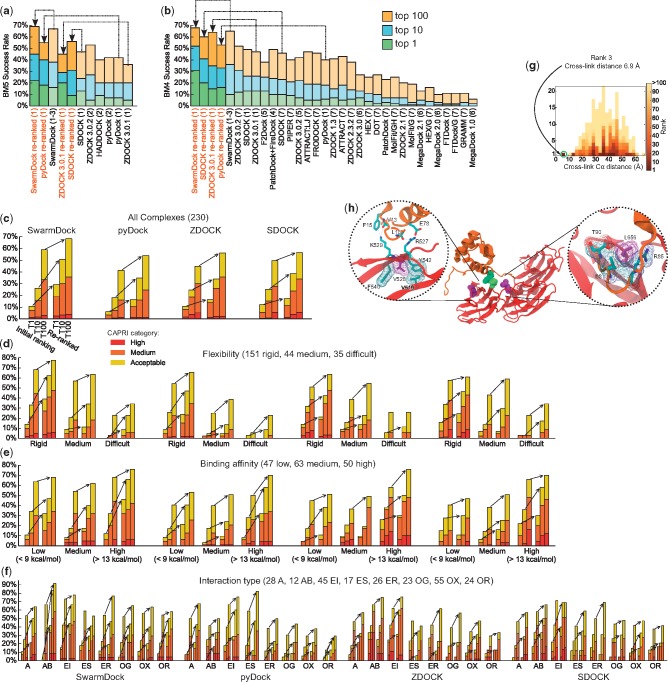
The top 1 and top 10 success rates for the whole docking pipeline for (**a**) the 55 new benchmark 5 complexes and (**b**) the 176 benchmark 4 complexes, using data from (1) this study, as original rankings (lighter colors) or using either the benchmark 5 complexes as external test set or multiple leave-many-out cross-validation with the benchmark 4 (dark colors), (2) Vreven *et al.*, 2015, (3) Torchala *et al.*, 2013, (4) Schneidman-Duhovny *et al.*, 2012, (5) Chowdhury *et al.*, 2013, (6) Ohue *et al.*, 2014, (7) Huang, 2015. Results from Huang use a slightly different definition of near-native. Arrows indicate the performance prior to re-ranking and following re-ranking. The left panels indicate (**c**) the performance according to CAPRI category, and when the data are partitioned by (**d**) flexibility, (**e**) affinity and (**f**) type categories. The docking results for the LCP2/FLNA interaction are shown in (**g**) and (**h**). Panel (**g**) shows a histogram of the distribution of C*α* distances between cross-linked residues K81 and K498 for all decoys. Decoys falling within each interval are ordered and colored according to rank. Panel (**h**) shows the docked structure, indicating K81 and K298 in green, and V528 and L656 in purple, with zoomed in and rotated regions indicating nearby interacting residues (Color version of this figure is available at *Bioinformatics* online.)

**Fig. 3:**
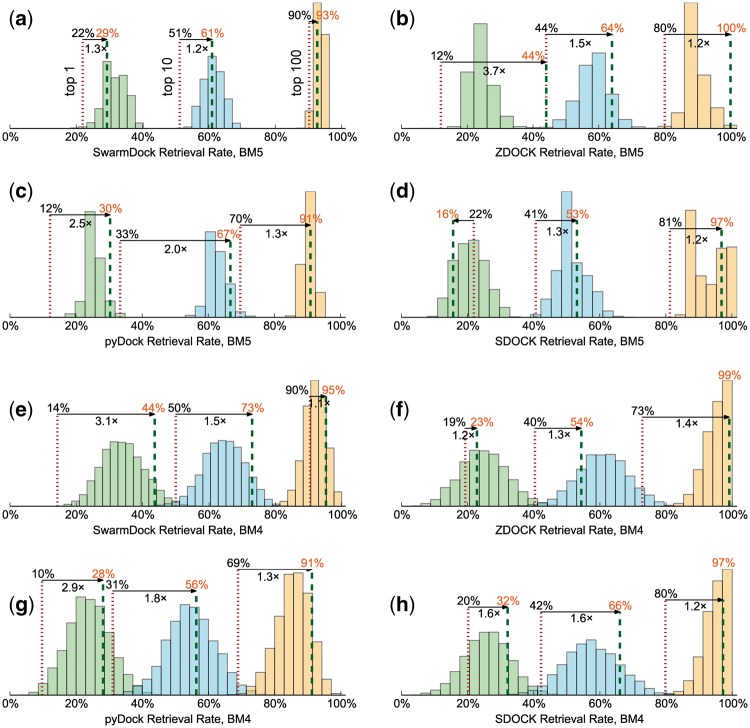
Each panel shows the top 1 (left), top 10 (centre) and top 100 (right) retrieval rates for the original (red dots) and consensus (green dashes) rankings, as well as the distributions for the ensembles of support vector machines when applied to the new complex in the benchmark 5 as external test set (**a–d**), and using multiple leave-many-out cross-validation of the benchmark 4 (**e–h**) (Color version of this figure is available at *Bioinformatics* online.)

During the training of the method there are two adjustable parameters, *c* (see Section 2.4) and *n*, the number of R-SVM models kept in step 4 above. As *c* is multiplicative, it was sampled logarithmically 50 times in the 10^−^^4^ to 10^3^ range inclusive. Steps 2 to 5 were performed 50 times concurrently for each sample of *c*. The value of *n* was also sampled 50 times (from *n* = 1 to *n* = 50) and tested concurrently in steps 4 to 5. Thus, results were gained for the entire 50 × 50 metaparameter grid. This systematic evaluation of parameter space reveals that the method is both insensitive to small changes and robust across a wide range of *c* and *n* values. For instance the SwarmDock benchmark 4 top 10 retrieval rate only varying between 67% and 74% ($\bar{x}=71$%, σ = 1.0%) in the *c* = [1.0,10^3^] and *n* = [5,40] grid. The success and retrieval rates in Figures 2 and 3, and Supplementary Table S1, correspond to *c* and *n* metaparameter values found by leave-one-out cross validation; for each complex in the test set, the sum of the scores (Section 2.5) of the remaining complexes was calculated for each point in the metaparameter grid, and the parameters corresponding to the highest score was applied to the complex (Supplementary Tables S3 and S4). The version implemented in the SwarmDock, ZDOCK and pyDock servers were re-trained with the entire benchmark 5 data using the mode average cross-validated *n* and *c* metaparameter pair obtained using the benchmark 4.0, reported in Supplementary Table S3. For the SwarmDock and pyDock servers, the decoys are the same as reported here. For the ZDOCK server the decoys were re-calculated using the most recent version of ZDOCK (3.0.2), for which tests using the BM4 data for training and new BM5 complexes for testing yielded similar results in terms of top 1/10/100 success rates and the identities of the complexes with high ranking near-native decoys.

### 2.4 The R-SVM method

The R-SVM method adopted here constructs a model which is trained to minimize the fraction of swapped pairs relative to a perfect ranking (Joachims, 2002). This is achieved by finding a weight vector in descriptor space, **w**, such that when the descriptor values ($d_{ik}$) for the decoys (*i*) of an interaction (*k*) are projected upon it to yield positions along that vector ($wd_{ik}$), the decoys can be ranked according to their positions. A perfect ranking would have the projected positions of all the decoys in the high quality CAPRI category greater than those in the medium category, which in turn are greater than those in the acceptable category, which are greater than those in the incorrect category. Formally, for all pairs of decoys (*i*, *j*) not in the same CAPRI category, where decoy *i* is closer to the native structure than *j*, the inequality $wd_{ik}>wd_{jk}$ would be satisfied. While in theory it may be possible for many weight vectors to achieve a perfect ranking, and thus choose **w** that maximizes the margin by which the CAPRI categories are separated by maximizing $2/||w|{|}^{2}$ (Vapnik, 1998), in reality the different CAPRI categories are not perfectly separable for all complexes. To account for this, non-negative slack variables (ξ_*ijk*_) can be introduced into each inequality, the sum of which can be minimized to bring the ranking as close as possible to a perfect ranking:

(1) $$wd_{ik}\geq wd_{jk}+1-\xi_{ijk}\;\text{where}\;\xi_{ijk}\geq 0\;\;\text{for}\;\;\;\text{all}\;\;\;k$$

Thus, the problem of getting as close as possible to a perfect ranking becomes one of simultaneously minimizing the square norm of **w** in order to maximize the soft margin, and minimizing the slack variables ξ_*ijk*_:

(2) $$\underset{w\in R^{91},\xi_{ijk}\in R_{\geq 0}}{\text{arg}\;\;\text{min}}(\frac{1}{2}||w|{|}^{2}+c\sum \xi_{ijk})\;\;\text{subject}\;\;\text{to}\;\;\;(1)$$

The *c* parameter controls the trade-off between the two terms being minimized in Equation 2. A large *c* puts the focus onto getting closest to perfect rankings on the training examples, while a small *c* puts more emphasis on maximizing the separation between CAPRI categories, even at the expense of misranking some decoys. The optimal choice of *c*, and the range across which it produces models that generalize well on unseen data, depends upon the training data. For instance proteins can often form long-lived associations via alternative binding sites or binding modes that are not evident in the crystal structure, and thus a correct binding mode may be misclassified as incorrect in the training data. By balancing *c*, it is possible for the R-SVM to find a weight vector that permits this misclassification rather than force it to be ranked highly, to the detriment of the confidence in which the majority of correctly classified poses are ranked.

The first constraint in Equation 1 can be rearranged to $w(d_{ik}-d_{jk})\geq 1-\xi_{ijk}$, which shows that the above ranking procedure is equivalent to an SVM classifier trained on the pairs of decoys for which an inequality is specified, with features corresponding to the difference between the individual features of the two decoys. Thus, the weight vector from such a classifier can be used to rank the decoys in the model selection and test sets. The optimization of Equation 2 was performed using the n-slack algorithm with shrinking heuristics described by Joachims (2005), through the SVM^rank^ program (Joachims, 2006). The average components of the weight vectors for top scoring models are shown in Supplementary Table S5.

### 2.5 Scoring R-SVM models

Each R-SVM model is evaluated by its total score *S*, the sum of individual scores for each of the *n_t_* complexes in the model selection set, *s_i_*, compared to the mean score for that complex across the R-SVM ensemble, $\bar{s_{i}}$:

(3) $$S=\sum_{i=1}^{n_{t}}\left(s_{i}-\bar{s_{i}}\right)$$

By taking the score relative to the mean, the total score reduces biases in the model selection set by preferentially favouring R-SVM models which perform well on difficult complexes, those which the other models struggle to perform well on, and disfavours models which perform poorly on easy complexes, those which the other models do perform well on.

For calculating the individual scores of a complex, the decoys are first clustered at 3.5 Å (see above). The clusters, the number of which we denote as *n_c_*, are ranked by the score of their top scoring decoy. The overall rank for the complex, *r*, is the rank of the best ranked cluster for which the top ranked decoy within that cluster is of acceptable quality or better. The scores are then calculated as:

(4) $$s_{i}=\frac{\;{\text{log}}_{10}(n_{c})-{\text{log}}_{10}(r)}{\;{\text{log}}_{10}(n_{c})}$$

This score can range from 0, if only the last cluster has a top ranked decoy that is not incorrect, to 1, where the top scoring decoy of the top ranked cluster is not incorrect. If no acceptable or better solutions appear as top ranked decoys within any cluster, *s_i_* is set to zero. The logarithmic form gives greater importance to higher ranks such that the increase in *s_i_* in going from rank 11 to rank 1 is far greater than going from, say, rank 411 to 401.

### 2.6 Applying the method with Schulze ranking

To apply the model to the test sets or new docking cases, the physicochemical features are calculated for each decoy and transformed to z-scores using the previously determined mean and σ values. The decoys are ranked using each of the *n* selected models in the R-SVM ensemble, by their order when projected onto the R-SVM weight vector line in descriptor space (Section 2.4). Each of these rankings is combined using the Schulze electoral voting system (Schulze, 2011). Firstly, a complete digraph is constructed in which each node corresponds to a decoy, and the edge weights indicate the number of times the tail node decoy is ranked higher than the head node decoy. This graph is then used to find the strongest paths between all ordered pairs of decoys, (*a*, *b*), where path strength corresponds to the minimum edge weight in a directed path originating at *a* and terminating at *b*. To clarify this point, the strength of any given path between the decoys corresponds to the smallest weight of any edge within the path. The strongest path is the path, out of all possible paths, that gives the highest strength. The identification of the strongest path is achieved using a dynamic programming approach (Schulze, 2011). Using this information, decoy *a* is ranked higher than decoy *b* if the strength of the strongest path of (*a*, *b*) is greater than that of (*b*, *a*). As preferences are transitive, a consensus ranking follows directly from the pairwise rankings.

## 3 Results and discussion

### 3.1 Validation and benchmarking

To validate the method, we trained the models using complexes in the protein–protein docking benchmark 4 (Hwang *et al.*, 2010), and evaluated the ability to retrieve near-native solutions using the new complexes added in the benchmark 5 (Vreven *et al.*, 2015) as an external validation set (Fig. 3a–d). Of the complexes for which a near-native solution could be found, a near-native structure was identified as the top ranked solution in 12–22% of the interactions prior to re-ranking, which increased to 16–44% using our approach. Similarly, retrieval in the top 10 increases from 33–51%, to 50–67% and top 100 improves from 70–90% to 91–100%, indicating that sampling becomes the limiting factor in obtaining a top 100 near-native solution within our scoring scheme. Considering this improvement in the context of the whole docking pipeline, which includes complexes for which no near-natives are sampled (Fig. 2a and Supplementary Table S1), this corresponds to top 1 success rates up to 22% for all 55 complexes in the benchmark 5 update, top 10 success rates up to 45%, and top 100 success rates up to 69%.

We also applied the method to the original complexes in the docking benchmark 4 using multiple leave-many-out cross-validation (Fig. 3e–h). A quarter of the complexes were left out at random from the training set for each of the R-SVM models, and for each complex the Schulze re-ranking only combined the models for which the complex was omitted from the training. We see improvements of 10–20% to 24–42%, 31–50% to 51–68% and 69–90% to 89–100% respectively for the top 1/10/100 retrieval rates. For SwarmDock, this corresponds to top 1/10/100 success rates of 31%, 52% and 68% respectively, when considering all 176 complex in the benchmark 4 (Fig. 2b and Supplementary Table S1). On both benchmarks, a large improvement can be attributed to the R-SVMs which, when combined using Schulze ranking, typically performing as good as or better than the average R-SVM model on its own. For all four docking protocols, the method yields a significantly better ranking of near-native solution (*P*≪0.01, Wilcoxon signed-rank test), and large improvements compared to other methods reported in the literature (Fig. 2a,b and Supplementary Table S1).

The combined results for the two benchmarks, and across the different CAPRI quality categories, are shown in Figure 2c. A strong improvement in success rates is seen for the acceptable and medium categories, although there are too few data to make conclusions regarding the high quality category. Further, the improvements in results apply across all categories of flexibility, binding affinity or biological role (Fig. 2d–f). As well as increasing the rank of near-native decoys, the method also improves the quality of the decoys at the top of the ranking, even when a high ranking near-native could be found in the initial rankings; considering the complexes for which a near-native could be found in the top 100 before and after re-ranking, the top-ranked near-native was of a higher CAPRI class for 20 complexes in the re-ranked list using the SwarmDock structures, compared to 13 that were higher in the initial rankings. For pyDock, ZDOCK and SDOCK these figures are 14 and 3, 22 and 7 and 22 and 6 respectively. This also holds when considering only the top 10 decoys, with values of 8 and 5, 6 and 2, 10 and 4, and 7 and 3 respectively.

### 3.2 Application to LCP2/FLNA interaction

To further validate the method, we investigated the interaction between the sterile alpha motif (SAM) domain of lymphocyte cytosolic protein 2 (LCP2, pdb 2EAP) with filamin A (FLNA, pdb 4M9P). This interaction was initially predicted based on co-expression, co-location, shared binding partners, and co-occurrence of post-translational modifications (McDowall *et al.*, 2009), and was recently confirmed by cross-linking mass spectrometry (Liu *et al.*, 2015). Using SwarmDock without distance restraints, we generated a single structure consistent with length and steric requirements of the observed K81-K498 disuccinimidyl sulfoxide cross-link at the periphery of the binding site (Kao *et al.*, 2011), with a K81-K498 Cα–Cα distance of 6.9 Å (Fig. 2g–h). This structure is ranked 3rd using the presented method, compared to a rank of 7 using the standard protocol from the SwarmDock server. FLNA deactivation is associated with periventricular heterotopia, an X-linked dominant disorder in females which is hemizygous lethal in males, that can also arise sporadically from partially deactivating missense mutations in FLNA. These mutations appear either in the first calponin homology domain, which binds actin, or in uncharacterized 3rd (V528M) (Kakita *et al.*, 2002) or 4th (L656F) (Sheen *et al.*, 2001) Ig-like domains. Despite V528 and L656 being 18 Å apart, both residues participate in the predicted interaction, either via direct packing of L656 with LCP2 T90 and I88, or as V528 acting to support its adjacent residues R527 and L529, of which the former forms a salt bridge with LCP2 E78, and the latter forms a cation-*π* interaction with F15 and a hydrogen bond with N82 and V13. This suggests that both mutations may interfere with communication between the cytoskelton and the phosphotyrosine signalling network via this interaction. Thus, our approach has allowed us to predict a low-energy structure of a previously uncharacterized complex, validated by chemical cross-linking and by reconciling the common pathology of spatially separate FLNA mutants which, at the same time, establishes a link between the LCP2/FLNA interaction and periventricular heterotopia.

### 3.3 Web server implementations

We have implemented the method in the following web servers:

- SwarmDock: As an option available at http://bmm.crick.ac.uk/∼SwarmDock/ (Torchala *et al.*, 2013)
- pyDockWeb: As a separate server to re-rank results generated with the pyDockWeb server, available at http://life.bsc.es/pid/pydockrescoring/ (Jiménez-García *et al.*, 2013)
- ZDOCK: As an option available at http://zdock.umassmed.edu/

Computation times are considerably longer than for a standard docking run due to the cost of the descriptor calculations; tests on the SwarmDock server between two proteins of around 250 residues typically take 11 h from submission. On all three servers we aim to return results within a day depending upon server loads.

## 4 Conclusion

In conclusion, integrating biophysical functions using methods originally developed for information retrieval and electoral voting provides a powerful method for enhancing the atomic modelling of protein complexes in a way that is tailored to the technique used to generate the models, resulting in both a better ranking of near-native decoys and an improvement in the quality of high-ranking near-native decoys.

## Supplementary Material

Supplementary Data
