# Supplementary material for: IRaPPA: information retrieval based integration of biophysical models for protein assembly selection
Source: Bioinformatics. 2017 Feb 14;33(12):1806–13. doi: 10.1093/bioinformatics/btx068 (PMC5783285; doi:10.1093/bioinformatics/btx068)
Supplement: Supplementary Data [file btx068_supp.pdf]

Supplementary Material for "IRaPPA: Information retrieval based integration of biophysical models for protein assembly selection"

Table 1: Docking success rates in top 1, top 10 and top 100, for the four docking pipelines before and after re-ranking, and a comparison to other docking protocols. For the BM5 update SwarmDock, pyDock, ZDOCK and SDOCK could find a near-native for 41, 33, 25 and 32 complexes respectively. For the BM4 these are 126, 103, 114 and 109.

**Method**	**BM5 update** n=55			**BM4** n=176		
	T1	T10	T100	T1	T10	T100
**SwarmDock +re-rank**[a]	12 (22%)	25 (45%)	38 (69%)	55 (31%)	92 (52%)	120 (68%)
SwarmDock[b,c,d]	9 (16%)	21 (38%)	37 (67%)	18 (10%)	63 (36%)	114 (65%)
**pyDock +re-rank**[a]	10 (18%)	22 (40%)	30 (55%)	29 (15%)	58 (33%)	94 (53%)
pyDock[b]	4 (7%)	11 (20%)	23 (41%)	10 (5%)	32 (18%)	71 (40%)
**ZDOCK 3.0.1+re-rank**[a]	11 (20%)	16 (29%)	25 (45%)	26 (15%)	62 (34%)	113 (64%)
ZDOCK 3.0.1[b]	3 (5%)	11 (20%)	20 (36%)	22 (12%)	46 (26%)	83 (47%)
**SDOCK +re-rank**[a]	5 (9%)	17 (31%)	31 (56%)	35 (20%)	72 (41%)	106 (60%)
SDOCK[b]	7 (12%)	13 (23%)	26 (47%)	22 (12%)	46 (26%)	87 (49%)
ZDOCK 3.0.2[c]	3 (5%)	15 (27%)	29 (52%)	-	-	-
pyDock[c]	4 (7%)	11 (20%)	23 (41%)	-	-	-
HADDOCK[c]	5 (9%)	11 (20%)	22 (40%)	-	-	-
PatchDock+FireDock[e]	-	-	-	- (10%)	- (24%)	- (49%)
F^2Dock[f]	-	-	-	22 (13%)	44 (25%)	65 (38%)
ZDOCK 3.0.2[f]	-	-	-	13 (7%)	38 (22%)	73 (42%)
MegaDock 1.0[g]	-	-	-	0 (0%)	4 (2%)	9 (6%)
MegaDock 2.0[g]	-	-	-	2 (1%)	5 (3%)	17 (10%)
MegaDock 2.1[g]	-	-	-	2 (1%)	8 (5%)	21 (13%)
ZDOCK 3.0[g]	-	-	-	12 (7%)	21 (13%)	48 (27%)
ZDOCK 3.0.2[h]	-	-	-	21 (12%)	54 (31%)	92 (52%)
SDOCK[h]	-	-	-	18 (10%)	40 (23%)	81 (46%)
PIPER[h]	-	-	-	15 (9%)	37 (21%)	70 (40%)
FRODOCK[h]	-	-	-	9 (5%)	34 (19%)	81 (46%)
ATTRACT:LJ[h]	-	-	-	9 (5%)	33 (19%)	83 (47%)
ATTRACT[h]	-	-	-	9 (5%)	32 (18%)	75 (43%)
ZDOCK 1.3[h]	-	-	-	12 (7%)	27 (15%)	73 (41%)
ZDOCK 2.3.2[h]	-	-	-	11 (6%)	25 (14%)	67 (38%)
HEX[h]	-	-	-	7 (4%)	19 (11%)	44 (25%)
DOT[h]	-	-	-	3 (2%)	17 (10%)	47 (27%)
PatchDock[h]	-	-	-	6 (3%)	13 (7%)	40 (23%)
MolFit/GH[h]	-	-	-	3 (2%)	13 (7%)	44 (25%)
ZDOCK 2.1[h]	-	-	-	2 (1%)	13 (7%)	36 (20%)
HEX/G[h]	-	-	-	0 (0%)	7 (4%)	29 (16%)
MolFit/G[h]	-	-	-	2 (1%)	5 (3%)	33 (19%)
GRAMM[h]	-	-	-	0 (0%)	5 (3%)	19 (11%)
FTDock/G[h]	-	-	-	1 (1%)	3 (2%)	20 (11%)
FTDock[h]	-	-	-	1 (1%)	3 (2%)	19 (11%)

[a]Re-ranked using IRaPPA. BM5 complexes are used as external validation set, and BM4 results are cross-validated.

[b]Results prior to re-ranking on the same decoy set.

[c]Results from Vreven *et al.* (2015).

[d]Results from Torchala *et al.* (2013).

[e]Results from Schneidman-Duhovny *et al.* (2012).

[f]Results from Chowdhury *et al.* (2013).

[g]Results from Ohue *et al.* (2014).

[h]Results from Huang (2015), where a slightly different definition of near-native is used.

Table 2: Descriptor list. For more details please see Moal *et al.* (2015)

#	ID	Type	Reference
1	CP_BFKV	Statistical potential	Feng *et al.* (2010)
2	CP_BL	Statistical potential	Feng *et al.* (2010)
3	CP_BT	Statistical potential	Feng *et al.* (2010)
4	CP_GKS	Statistical potential	Feng *et al.* (2010)
5	CP_HLPL	Statistical potential	Feng *et al.* (2010)
6	CP_MJPL	Statistical potential	Feng *et al.* (2010)
7	CP_MJ3h	Statistical potential	Feng *et al.* (2010)
8	CP_MJ2h	Statistical potential	Feng *et al.* (2010)
9	CP_MJ1	Statistical potential	Feng *et al.* (2010)
10	CP_MJ2	Statistical potential	Feng *et al.* (2010)
11	CP_MSBM	Statistical potential	Feng *et al.* (2010)
12	CP_MS	Statistical potential	Feng *et al.* (2010)
13	CP_Qa	Statistical potential	Feng *et al.* (2010)
14	CP_Qm	Statistical potential	Feng *et al.* (2010)
15	CP_Qp	Statistical potential	Feng *et al.* (2010)
16	CP_RO	Statistical potential	Feng *et al.* (2010)
17	CP_SKOb	Statistical potential	Feng *et al.* (2010)
18	CP_SKOa	Statistical potential	Feng *et al.* (2010)
19	CP_SJKG	Statistical potential	Feng *et al.* (2010)
20	CP_TD	Statistical potential	Feng *et al.* (2010)
21	CP_TEl	Statistical potential	Feng *et al.* (2010)
22	CP_TEs	Statistical potential	Feng *et al.* (2010)
23	CP_TS	Statistical potential	Feng *et al.* (2010)
24	CP_VD	Statistical potential	Feng *et al.* (2010)
25	CP_SKOIP	Statistical potential	Lu *et al.* (2003)
26	AP_DCOMPLEX	Statistical potential	Liu *et al.* (2004)
27	AP_dDFIRE	Statistical potential	Yang & Zhou (2008*a*)
28	AP_DFIRE2	Statistical potential	Yang & Zhou (2008*b*)
29	CP_RMFCEN1	Statistical potential	Rajgaria *et al.* (2008)
30	CP_RMFCEN2	Statistical potential	Rajgaria *et al.* (2008)
31	CP_RMFCA	Statistical potential	Rajgaria *et al.* (2006)
32	CP_TB	Statistical potential	Tobi & Bahar (2006)
33	CP_TSC	Statistical potential	Tobi (2010)
34	AP_T1	Statistical potential	Tobi (2010)
35	AP_T2	Statistical potential	Tobi (2010)
36	AP_DOPE	Statistical potential	Shen & Sali (2006)
37	AP_DOPE_HR	Statistical potential	Shen & Sali (2006)
38	AP_ACE	Statistical potential	Andrusier *et al.* (2007)
39	INSIDE	Geometric term	Andrusier *et al.* (2007)
40	HBOND	Hydrogen-bonding	Andrusier *et al.* (2007)
41	PI_PI	Aromatic interactions	Andrusier *et al.* (2007)
42	CAT_PI	Cation-pi interactions	Andrusier *et al.* (2007)
43	ALIPH	Aliphatic interactions	Andrusier *et al.* (2007)
44	ZRANK	Composite score	Pierce & Weng (2007)
45	ZRANK2	Composite score	Pierce & Weng (2008)
46	ROT_S	Rotational entropy	Brooks *et al.* (2009)
47	TRANS_S	Translational entropy	Brooks *et al.* (2009)
48	FA_ATR	VDW attraction	Chaudhury *et al.* (2010)
49	FA_REP	VDW repulsion	Chaudhury *et al.* (2010)
50	LK_SOLV	Solvation	Chaudhury *et al.* (2010)
51	FA_PP	Statistical potential	Chaudhury *et al.* (2010)
52	CG_VDW	VDW energy	Chaudhury *et al.* (2010)
53	CG_PP	Statistical potential	Chaudhury *et al.* (2010)
54	CG_ENV	Statistical potential	Chaudhury *et al.* (2010)
55	CG_BETA	Statistical potential	Chaudhury *et al.* (2010)
56	HBOND2	Hydrogen-bonding	Chaudhury *et al.* (2010)
57	ROSETTA	Composite score	Chaudhury *et al.* (2010)
58	AA_PROP	Statistical potential	Chaudhury *et al.* (2010)
59	ROSETTADOCK	Composite score	Chaudhury *et al.* (2010)
60	NHB	# hydrogen bonds	Chaudhury *et al.* (2010)
61	AP_OPUS_PSP	Statistical potential	Lu *et al.* (2008)
62	AP_GEOMETRIC	Statistical potential	Unpublished (Li, X. and Liang, J.)
63	AP_DARS	Statistical potential	Chuang *et al.* (2008)
64	AP_URS	Statistical potential	Chuang *et al.* (2008)
65	AP_MPS	Statistical potential	Chuang *et al.* (2008)
66	AP_W1	Statistical potential	Mintseris *et al.* (2007)
67	CP_D1	Statistical potential	Liu & Vakser (2011)
68	AP_calRW	Statistical potential	Zhang & Zhang (2010)
69	AP_calRWp	Statistical potential	Zhang & Zhang (2010)
70	AP_GOAP_ALL	Statistical potential	Zhou & Skolnick (2011)
71	AP_GOAP_DF	Statistical potential	Zhou & Skolnick (2011)
72	AP_GOAP_G	Statistical potential	Zhou & Skolnick (2011)

#	ID	Type	Reference
73	AP_PISA	Composite score	Viswanath *et al.* (2013)
74	FIREDOCK	Composite score	Andrusier *et al.* (2007)
75	FIREDOCK_AB	Composite score	Andrusier *et al.* (2007)
76	FIREDOCK_EI	Composite score	Andrusier *et al.* (2007)
77	CP_PIE	Composite score	Ravikant & Elber (2010)
78	CP_DDG_U	Empirical potential	Moal & Fernandez-Recio (2013)
79	CP_DDG_W	Empirical potential	Moal & Fernandez-Recio (2013)
80	AP_DDG_U	Empirical potential	Moal & Fernandez-Recio (2013)
81	AP_DDG_W	Empirical potential	Moal & Fernandez-Recio (2013)
82	DDG_V	Empirical potential	Moal *et al.* (2015)
83	Clust_0.3	Cluster size	
84	Clust_0.35	Cluster size	
85	Clust_0.4	Cluster size	
86	Clust_0.45	Cluster size	
87	Clust_0.5	Cluster size	
88	Clust_0.55	Cluster size	
89	Clust_0.6	Cluster size	
90	Clust_0.65	Cluster size	

Table 3: Highest scoring n and c parameters in each iteration of the leave-one-out cross-validation loop for Benchmark 4.0 complexes, plus the rank of the highest-ranking near-native decoy when ranked using those parameters. The mode average n and c metaparameter pair are 38 and 72.0, 50 and 0.518, 22 and 72.0, and 43 and 0.373 for SwarmDock, pyDock, ZDOCK and SDOCK respectively. For pyDock the $n = 50$ value is the largest on the metaparameter grid, which indicates that further improvements may be gained by increasing this parameter further.

	SwarmDock			pyDock			ZDOCK			SDOCK		
Complex	n	c	*rank*	n	c	*rank*	n	c	*rank*	n	c	*rank*
1B6C	38	7.20×10^{1}	1	50	5.18×10^{-1}	1	22	7.20×10^{1}	1	43	3.73×10^{-1}	1
1BVN	38	7.20×10^{1}	1	50	5.18×10^{-1}	1	22	7.20×10^{1}	1	43	3.73×10^{-1}	1
1FSK	38	7.20×10^{1}	1	50	5.18×10^{-1}	1	22	7.20×10^{1}	1	43	3.73×10^{-1}	1
1JTG	38	7.20×10^{1}	1	50	5.18×10^{-1}	1	22	7.20×10^{1}	1	43	3.73×10^{-1}	1
1K74	38	7.20×10^{1}	1	50	5.18×10^{-1}	1	22	7.20×10^{1}	1	43	3.73×10^{-1}	1
1KXP	38	7.20×10^{1}	1	50	5.18×10^{-1}	1	22	7.20×10^{1}	1	43	3.73×10^{-1}	1
1UDI	38	7.20×10^{1}	1	50	5.18×10^{-1}	1	22	7.20×10^{1}	1	43	3.73×10^{-1}	1
2OUL	38	7.20×10^{1}	1	50	5.18×10^{-1}	1	22	7.20×10^{1}	1	43	3.73×10^{-1}	1
2VDB	38	7.20×10^{1}	1	50	5.18×10^{-1}	1	22	7.20×10^{1}	1	43	3.73×10^{-1}	1
4CPA	38	7.20×10^{1}	1	50	5.18×10^{-1}	1	22	7.20×10^{1}	1	43	3.73×10^{-1}	1
1E6E	38	7.20×10^{1}	1	50	5.18×10^{-1}	1	47	5.18×10^{-2}	2	43	3.73×10^{-1}	1
1GPW	38	7.20×10^{1}	1	50	5.18×10^{-1}	1	22	7.20×10^{1}	2	43	3.73×10^{-1}	1
1IQD	38	7.20×10^{1}	1	50	5.18×10^{-1}	2	22	7.20×10^{1}	1	43	3.73×10^{-1}	1
1N8O	38	7.20×10^{1}	1	50	5.18×10^{-1}	1	22	7.20×10^{1}	2	43	3.73×10^{-1}	1
1PPE	38	7.20×10^{1}	2	50	5.18×10^{-1}	1	22	7.20×10^{1}	1	43	3.73×10^{-1}	1
1EWY	38	7.20×10^{1}	1	50	5.18×10^{-1}	3	22	7.20×10^{1}	1	43	3.73×10^{-1}	1
1OYV	38	7.20×10^{1}	1	50	5.18×10^{-1}	3	22	7.20×10^{1}	1	43	3.73×10^{-1}	1
1TMQ	38	7.20×10^{1}	1	50	5.18×10^{-1}	1	22	7.20×10^{1}	3	43	3.73×10^{-1}	1
2B42	38	7.20×10^{1}	2	50	5.18×10^{-1}	1	47	5.18×10^{-2}	2	43	3.73×10^{-1}	1
1R0R	38	7.20×10^{1}	1	50	5.18×10^{-1}	3	22	7.20×10^{1}	2	43	3.73×10^{-1}	1
7CEI	38	7.20×10^{1}	2	50	5.18×10^{-1}	1	22	7.20×10^{1}	2	43	3.73×10^{-1}	2
1CLV	38	7.20×10^{1}	1	50	5.18×10^{-1}	4	22	7.20×10^{1}	1	43	3.73×10^{-1}	2
2FD6	38	7.20×10^{1}	2	50	5.18×10^{-1}	3	22	7.20×10^{1}	1	43	3.73×10^{-1}	2
1MAH	38	7.20×10^{1}	1	49	1.39×10^{-1}	4	22	7.20×10^{1}	2	43	3.73×10^{-1}	2
2JEL	38	7.20×10^{1}	2	50	5.18×10^{-1}	1	22	7.20×10^{1}	5	43	3.73×10^{-1}	1
1FLE	38	7.20×10^{1}	5	50	5.18×10^{-1}	2	22	7.20×10^{1}	2	43	3.73×10^{-1}	1
2AYO	38	7.20×10^{1}	5	50	5.18×10^{-1}	1	47	5.18×10^{-2}	2	43	3.73×10^{-1}	2
1AY7	38	7.20×10^{1}	1	50	5.18×10^{-1}	5	22	7.20×10^{1}	2	43	3.73×10^{-1}	4
1J2J	38	7.20×10^{1}	1	50	5.18×10^{-1}	5	22	7.20×10^{1}	5	43	3.73×10^{-1}	3
2I25	38	7.20×10^{1}	5	50	5.18×10^{-1}	4	22	7.20×10^{1}	2	43	3.73×10^{-1}	3
1FFW	38	7.20×10^{1}	5	50	5.18×10^{-1}	5	22	7.20×10^{1}	4	43	3.73×10^{-1}	1
1ML0	38	7.20×10^{1}	1	50	5.18×10^{-1}	12	22	7.20×10^{1}	1	43	3.73×10^{-1}	1
1Z0K	38	7.20×10^{1}	1	50	5.18×10^{-1}	1	47	5.18×10^{-2}	2	43	3.73×10^{-1}	11
2BTF	38	7.20×10^{1}	1	50	5.18×10^{-1}	10	47	5.18×10^{-2}	3	43	3.73×10^{-1}	4
1E96	38	7.20×10^{1}	1	50	5.18×10^{-1}	1	22	7.20×10^{1}	4	43	3.73×10^{-1}	13
1GL1	38	7.20×10^{1}	1	50	5.18×10^{-1}	1	22	7.20×10^{1}	11	43	3.73×10^{-1}	6
1WEJ	38	7.20×10^{1}	1	50	5.18×10^{-1}	20	22	7.20×10^{1}	3	43	3.73×10^{-1}	2
1CGI	38	7.20×10^{1}	2	50	5.18×10^{-1}	23	22	7.20×10^{1}	1	43	3.73×10^{-1}	2
1XD3	38	7.20×10^{1}	1	50	5.18×10^{-1}	1	22	7.20×10^{1}	1	43	3.73×10^{-1}	29
2G77	38	7.20×10^{1}	1	50	5.18×10^{-1}	5	22	7.20×10^{1}	17	43	3.73×10^{-1}	9
3SGQ	38	7.20×10^{1}	3	50	5.18×10^{-1}	16	47	5.18×10^{-2}	6	43	3.73×10^{-1}	8
2HLE	38	7.20×10^{1}	1	50	5.18×10^{-1}	7	22	7.20×10^{1}	16	43	3.73×10^{-1}	13
2IDO	38	7.20×10^{1}	6	50	5.18×10^{-1}	13	22	7.20×10^{1}	4	43	3.73×10^{-1}	15
1OC0	38	7.20×10^{1}	4	50	5.18×10^{-1}	26	22	7.20×10^{1}	15	43	3.73×10^{-1}	10
1AVX	38	7.20×10^{1}	51	50	5.18×10^{-1}	2	22	7.20×10^{1}	2	43	3.73×10^{-1}	1
1BUH	38	7.20×10^{1}	1	50	5.18×10^{-1}	15	22	7.20×10^{1}	33	43	3.73×10^{-1}	9
1E6J	38	7.20×10^{1}	13	49	1.00×10^{0}	2	22	7.20×10^{1}	31	43	3.73×10^{-1}	12
1WDW	38	7.20×10^{1}	1	50	5.18×10^{-1}	34	22	7.20×10^{1}	22	14	1.39×10^{1}	8
1WQ1	38	7.20×10^{1}	13	50	5.18×10^{-1}	12	47	5.18×10^{-2}	36	43	3.73×10^{-1}	9
1JK9	38	7.20×10^{1}	6	50	5.18×10^{-1}	30	22	7.20×10^{1}	35	43	3.73×10^{-1}	7
2HRK	38	7.20×10^{1}	9	50	5.18×10^{-1}	4	22	7.20×10^{1}	29	43	3.73×10^{-1}	57
2I9B	38	7.20×10^{1}	1	50	5.18×10^{-1}	8	22	7.20×10^{1}	29	43	3.73×10^{-1}	78
1R6Q	38	7.20×10^{1}	1	50	5.18×10^{-1}	32	47	5.18×10^{-2}	32	43	3.73×10^{-1}	61
1GLA	38	7.20×10^{1}	5	50	5.18×10^{-1}	30	26	3.73×10^{-1}	53	43	3.73×10^{-1}	47
1H9D	38	7.20×10^{1}	11	50	5.18×10^{-1}	32	22	7.20×10^{1}	11	43	3.73×10^{-1}	94
2MTA	38	7.20×10^{1}	139	50	5.18×10^{-1}	2	22	7.20×10^{1}	8	43	3.73×10^{-1}	4
1VFB	38	7.20×10^{1}	2	50	5.18×10^{-1}	67	47	5.18×10^{-2}	61	43	3.73×10^{-1}	48
1I9R	38	7.20×10^{1}	4	50	5.18×10^{-1}	23	22	7.20×10^{1}	139	43	3.73×10^{-1}	30
1ZM4	38	7.20×10^{1}	213	50	5.18×10^{-1}	27	28	2.68×10^{2}	7	43	3.73×10^{-1}	28
1DFJ	38	7.20×10^{1}	1	-	-	-	22	7.20×10^{1}	1	43	3.73×10^{-1}	1
1KXQ	38	7.20×10^{1}	1	-	-	-	22	7.20×10^{1}	1	43	3.73×10^{-1}	1
1RV6	38	7.20×10^{1}	1	-	-	-	22	7.20×10^{1}	1	43	3.73×10^{-1}	1
2CFH	38	7.20×10^{1}	1	-	-	-	22	7.20×10^{1}	1	43	3.73×10^{-1}	1
2SNI	-	-	-	50	5.18×10^{-1}	1	22	7.20×10^{1}	1	43	3.73×10^{-1}	1

	SwarmDock			pyDock			ZDOCK			SDOCK		
Complex	*n*	*c*	*rank*	*n*	*c*	*rank*	*n*	*c*	*rank*	*n*	*c*	*rank*
1YVB	38	7.20×10^{1}	1	-	-	-	47	5.18×10^{-2}	3	43	3.73×10^{-1}	1
2SIC	-	-	-	50	5.18×10^{-1}	1	22	7.20×10^{1}	2	43	3.73×10^{-1}	2
2PCC	38	7.20×10^{1}	1	50	5.18×10^{-1}	1	15	7.20×10^{0}	5	-	-	-
1SYX	38	7.20×10^{1}	2	-	-	-	47	5.18×10^{-2}	3	43	3.73×10^{-1}	4
3D5S	38	7.20×10^{1}	1	-	-	-	22	7.20×10^{1}	7	43	3.73×10^{-1}	4
1F34	38	7.20×10^{1}	2	50	5.18×10^{-1}	10	22	7.20×10^{1}	1	-	-	-
1AZS	38	7.20×10^{1}	1	-	-	-	22	7.20×10^{1}	3	43	3.73×10^{-1}	11
1I2M	38	7.20×10^{1}	1	-	-	-	47	5.18×10^{-2}	13	43	3.73×10^{-1}	8
1LFD	38	7.20×10^{1}	6	50	5.18×10^{-1}	10	-	-	-	43	3.73×10^{-1}	6
1US7	38	7.20×10^{1}	10	50	5.18×10^{-1}	1	22	7.20×10^{1}	16	-	-	-
2HMI	38	7.20×10^{1}	2	-	-	-	47	5.18×10^{-2}	18	43	3.73×10^{-1}	7
2ABZ	-	-	-	35	5.18×10^{0}	15	22	7.20×10^{1}	14	43	3.73×10^{-1}	1
1RLB	-	-	-	50	5.18×10^{-1}	28	22	7.20×10^{1}	3	43	3.73×10^{-1}	1
1NCA	38	7.20×10^{1}	4	50	5.18×10^{-1}	10	47	5.18×10^{-2}	20	-	-	-
2UUY	-	-	-	50	5.18×10^{-1}	3	47	5.18×10^{-2}	30	43	3.73×10^{-1}	3
1ACB	-	-	-	50	5.18×10^{-1}	8	47	5.18×10^{-2}	27	43	3.73×10^{-1}	8
1K5D	38	7.20×10^{1}	2	50	5.18×10^{-1}	12	22	7.20×10^{1}	33	-	-	-
1MLC	-	-	-	50	5.18×10^{-1}	35	22	7.20×10^{1}	12	43	3.73×10^{-1}	3
1EAW	38	7.20×10^{1}	1	-	-	-	22	7.20×10^{1}	2	43	3.73×10^{-1}	54
1GXD	38	7.20×10^{1}	9	-	-	-	22	7.20×10^{1}	44	43	3.73×10^{-1}	9
1XQS	38	7.20×10^{1}	47	-	-	-	22	7.20×10^{1}	10	43	3.73×10^{-1}	5
1OPH	38	7.20×10^{1}	4	50	5.18×10^{-1}	36	47	5.18×10^{-2}	23	-	-	-
2NZ8	38	7.20×10^{1}	48	-	-	-	47	5.18×10^{-2}	15	43	3.73×10^{-1}	3
1GP2	-	-	-	50	5.18×10^{-1}	32	22	7.20×10^{1}	18	43	3.73×10^{-1}	19
1JWH	38	7.20×10^{1}	1	-	-	-	22	7.20×10^{1}	13	43	3.73×10^{-1}	56
1NW9	38	7.20×10^{1}	18	-	-	-	22	7.20×10^{1}	32	43	3.73×10^{-1}	23
2O8V	-	-	-	50	5.18×10^{-1}	33	22	7.20×10^{1}	28	43	3.73×10^{-1}	16
2OT3	-	-	-	50	5.18×10^{-1}	23	47	5.18×10^{-2}	38	43	3.73×10^{-1}	18
1BJ1	-	-	-	50	5.18×10^{-1}	27	22	7.20×10^{1}	10	43	3.73×10^{-1}	46
2H7V	-	-	-	50	5.18×10^{-1}	79	22	7.20×10^{1}	1	43	3.73×10^{-1}	6
1JZD	-	-	-	49	1.00×10^{0}	77	47	5.18×10^{-2}	8	43	3.73×10^{-1}	23
1HCF	38	7.20×10^{1}	1	-	-	-	22	7.20×10^{1}	29	43	3.73×10^{-1}	90
1HIA	38	7.20×10^{1}	86	50	5.18×10^{-1}	14	22	7.20×10^{1}	24	-	-	-
2B4J	38	7.20×10^{1}	22	50	5.18×10^{-1}	58	-	-	-	43	3.73×10^{-1}	50
1ATN	38	7.20×10^{1}	9	-	-	-	22	7.20×10^{1}	56	43	3.73×10^{-1}	73
1F51	38	7.20×10^{1}	3	50	5.18×10^{-1}	153	22	7.20×10^{1}	36	-	-	-
2HQS	38	7.20×10^{1}	131	50	5.18×10^{-1}	68	-	-	-	43	3.73×10^{-1}	39
1OFU	38	7.20×10^{1}	99	-	-	-	22	7.20×10^{1}	58	43	3.73×10^{-1}	108
1JIW	38	7.20×10^{1}	48	50	5.18×10^{-1}	183	22	7.20×10^{1}	37	-	-	-
1I4D	38	7.20×10^{1}	29	50	5.18×10^{-1}	275	22	7.20×10^{1}	74	-	-	-
1HE1	38	7.20×10^{1}	1	-	-	-	-	-	-	43	3.73×10^{-1}	1
1T6B	38	7.20×10^{1}	1	-	-	-	-	-	-	43	3.73×10^{-1}	1
1DE4	-	-	-	-	-	-	22	7.20×10^{1}	4	43	3.73×10^{-1}	3
1JPS	38	7.20×10^{1}	1	50	5.18×10^{-1}	8	-	-	-	-	-	-
1XU1	38	7.20×10^{1}	2	50	5.18×10^{-1}	9	-	-	-	-	-	-
9QFW	38	7.20×10^{1}	3	-	-	-	47	5.18×10^{-2}	12	43	3.73×10^{-1}	2
1AK4	-	-	-	50	5.18×10^{-1}	1	-	-	-	43	3.73×10^{-1}	17
1N2C	38	7.20×10^{1}	3	-	-	-	47	5.18×10^{-2}	16	-	-	-
1ZHI	-	-	-	-	-	-	47	5.18×10^{-2}	19	43	3.73×10^{-1}	6
2A5T	38	7.20×10^{1}	20	-	-	-	-	-	-	43	3.73×10^{-1}	5
1K4C	38	7.20×10^{1}	1	-	-	-	47	5.18×10^{-2}	26	-	-	-
2OOR	38	7.20×10^{1}	15	-	-	-	-	-	-	43	3.73×10^{-1}	13
2J0T	38	7.20×10^{1}	33	50	5.18×10^{-1}	9	-	-	-	-	-	-
1A2K	38	7.20×10^{1}	10	-	-	-	47	5.18×10^{-2}	39	-	-	-
1KKL	-	-	-	50	5.18×10^{-1}	2	-	-	-	43	3.73×10^{-1}	77
1KAC	38	7.20×10^{1}	8	-	-	-	-	-	-	43	3.73×10^{-1}	85
1DQJ	38	7.20×10^{1}	66	-	-	-	27	3.73×10^{2}	53	-	-	-
1BVK	-	-	-	50	5.18×10^{-1}	91	47	5.18×10^{-2}	34	-	-	-
2OOB	38	7.20×10^{1}	45	50	5.18×10^{-1}	80	-	-	-	-	-	-
2OZA	38	7.20×10^{1}	97	-	-	-	47	5.18×10^{-2}	28	-	-	-
1NSN	38	7.20×10^{1}	1	50	5.18×10^{-1}	139	-	-	-	-	-	-
1QFW	38	7.20×10^{1}	28	50	5.18×10^{-1}	136	-	-	-	-	-	-
2Z0E	38	7.20×10^{1}	48	-	-	-	-	-	-	43	3.73×10^{-1}	150
1FQJ	38	7.20×10^{1}	119	50	5.18×10^{-1}	206	-	-	-	-	-	-
1SBB	38	7.20×10^{1}	86	50	5.18×10^{-1}	245	-	-	-	-	-	-
1ZHH	-	-	-	35	5.18×10^{0}	241	-	-	-	41	3.73×10^{-1}	149
1AKJ	38	7.20×10^{1}	91	50	5.18×10^{-1}	311	-	-	-	-	-	-
1AHW	38	7.20×10^{1}	1	-	-	-	-	-	-	-	-	-
1D6R	38	7.20×10^{1}	1	-	-	-	-	-	-	-	-	-
1PVH	-	-	-	50	5.18×10^{-1}	1	-	-	-	-	-	-
1Z5Y	38	7.20×10^{1}	1	-	-	-	-	-	-	-	-	-
3BP8	38	7.20×10^{1}	2	-	-	-	-	-	-	-	-	-
1FCC	38	7.20×10^{1}	8	-	-	-	-	-	-	-	-	-

	SwarmDock			pyDock			ZDOCK			SDOCK		
Complex	n	c	$rank$	n	c	$rank$	n	c	$rank$	n	c	$rank$
1S1Q	38	7.20×10^1	8	-	-	-	-	-	-	-	-	-
1FQ1	38	7.20×10^1	14	-	-	-	-	-	-	-	-	-
1HE8	38	7.20×10^1	17	-	-	-	-	-	-	-	-	-
1GCQ	38	7.20×10^1	21	-	-	-	-	-	-	-	-	-
2AJF	-	-	-	-	-	-	22	7.20×10^1	24	-	-	-
1GRN	38	7.20×10^1	27	-	-	-	-	-	-	-	-	-
2A9K	-	-	-	50	5.18×10^{-1}	37	-	-	-	-	-	-
2O3B	-	-	-	50	5.18×10^{-1}	40	-	-	-	-	-	-
2VIS	38	7.20×10^1	40	-	-	-	-	-	-	-	-	-
1IJK	-	-	-	-	-	-	-	-	-	43	3.73×10^{-1}	47
1M10	-	-	-	-	-	-	-	-	-	43	3.73×10^{-1}	48
1EER	-	-	-	-	-	-	-	-	-	43	3.73×10^{-1}	51
1KTZ	-	-	-	-	-	-	22	7.20×10^1	56	-	-	-
3CPH	-	-	-	-	-	-	47	5.18×10^{-2}	56	-	-	-
1EZU	-	-	-	-	-	-	47	5.18×10^{-2}	62	-	-	-
1FC2	-	-	-	43	5.18×10^0	74	-	-	-	-	-	-
1EFN	-	-	-	50	5.18×10^{-1}	75	-	-	-	-	-	-
1JMO	38	7.20×10^1	95	-	-	-	-	-	-	-	-	-
1QA9	38	7.20×10^1	108	-	-	-	-	-	-	-	-	-
1MQ8	38	7.20×10^1	171	-	-	-	-	-	-	-	-	-

Table 4: Highest scoring n and c parameters in each iteration of the leave-one-out cross-validation loop for the new additions to the Benchmark 5.0, plus the rank of the highest-ranking near-native decoy when ranked using those parameters.

	SwarmDock			pyDock			ZDOCK			SDOCK		
Complex	n	c	*rank*	n	c	*rank*	n	c	*rank*	n	c	*rank*
3PC8	42	3.73×10^{-2}	1	32	7.20×10^{-2}	1	42	5.18×10^{2}	1	17	5.18×10^{-2}	1
4DN4	42	3.73×10^{-2}	1	32	7.20×10^{-2}	1	42	5.18×10^{2}	1	17	5.18×10^{-2}	2
4G6M	42	3.73×10^{-2}	1	32	7.20×10^{-2}	1	42	5.18×10^{2}	1	17	5.18×10^{-2}	2
3LVK	42	3.73×10^{-2}	1	32	7.20×10^{-2}	1	42	5.18×10^{2}	1	17	5.18×10^{-2}	4
2YVJ	21	2.68×10^{-3}	6	32	7.20×10^{-2}	8	42	5.18×10^{2}	1	17	5.18×10^{-2}	1
1JTD	42	3.73×10^{-2}	14	32	7.20×10^{-2}	1	42	5.18×10^{2}	1	17	5.18×10^{-2}	2
3BX7	21	2.68×10^{-3}	5	32	7.20×10^{-2}	14	42	5.18×10^{2}	1	17	5.18×10^{-2}	1
3F1P	42	3.73×10^{-2}	1	32	7.20×10^{-2}	9	42	5.18×10^{2}	6	17	5.18×10^{-2}	5
3A4S	42	3.73×10^{-2}	1	32	7.20×10^{-2}	5	42	5.18×10^{2}	2	3	2.68×10^{-2}	17
4G6J	21	2.68×10^{-3}	9	32	7.20×10^{-2}	6	42	5.18×10^{2}	2	17	5.18×10^{-2}	13
CP57	42	3.73×10^{-2}	1	25	7.20×10^{-2}	18	42	5.18×10^{2}	8	5	2.68×10^{-2}	9
3MXW	42	3.73×10^{-2}	1	32	7.20×10^{-2}	36	42	5.18×10^{2}	1	17	5.18×10^{-2}	1
3L5W	42	3.73×10^{-2}	7	31	7.20×10^{-2}	13	42	5.18×10^{2}	21	17	5.18×10^{-2}	9
3K75	42	2.68×10^{-3}	5	32	7.20×10^{-2}	2	42	5.18×10^{2}	13	17	5.18×10^{-2}	56
3P57	32	1.39×10^{-2}	15	32	7.20×10^{-2}	6	42	5.18×10^{2}	32	17	5.18×10^{-2}	58
3EOA	42	3.73×10^{-2}	73	32	7.20×10^{-2}	181	42	5.18×10^{2}	16	17	5.18×10^{-2}	55
3HMX	42	3.73×10^{-2}	2	32	7.20×10^{-2}	1	42	5.18×10^{2}	1	-	-	-
2X9A	44	3.73×10^{-2}	2	32	7.20×10^{-2}	1	-	-	-	17	5.18×10^{-2}	3
2A1A	42	3.73×10^{-2}	1	32	7.20×10^{-2}	3	-	-	-	26	5.18×10^{-2}	3
3VLB	42	3.73×10^{-2}	1	32	7.20×10^{-2}	7	-	-	-	17	5.18×10^{-2}	1
3DAW	42	3.73×10^{-2}	1	-	-	-	42	5.18×10^{2}	1	17	5.18×10^{-2}	12
3G6D	-	-	-	32	7.20×10^{-2}	1	42	5.18×10^{2}	12	17	5.18×10^{-2}	11
1EXB	42	3.73×10^{-2}	6	-	-	-	42	5.18×10^{2}	20	17	5.18×10^{-2}	14
2W9E	-	-	-	32	7.20×10^{-2}	1	42	5.18×10^{2}	14	17	5.18×10^{-2}	26
4H03	-	-	-	32	7.20×10^{-2}	3	42	5.18×10^{2}	82	17	5.18×10^{-2}	2
4FZA	-	-	-	32	7.20×10^{-2}	70	42	5.18×10^{2}	9	17	5.18×10^{-2}	35
4FQI	42	3.73×10^{-2}	8	32	7.20×10^{-2}	1	-	-	-	17	5.18×10^{-2}	129
2VXT	-	-	-	48	3.73×10^{-2}	4	42	5.18×10^{2}	1	-	-	-
1M27	21	2.68×10^{-3}	12	32	7.20×10^{-2}	5	-	-	-	-	-	-
3EO1	42	3.73×10^{-2}	10	-	-	-	-	-	-	17	5.18×10^{-2}	25
3H11	42	3.73×10^{-2}	38	-	-	-	-	-	-	17	5.18×10^{-2}	3
3L89	42	3.73×10^{-2}	1	-	-	-	42	5.18×10^{2}	42	-	-	-
3FN1	21	2.68×10^{-3}	17	-	-	-	-	-	-	17	5.18×10^{-2}	89
4IZ7	42	3.73×10^{-2}	22	-	-	-	-	-	-	5	2.68×10^{-2}	91
2GTP	21	2.68×10^{-3}	46	32	7.20×10^{-2}	75	-	-	-	-	-	-
4LW4	42	3.73×10^{-2}	138	48	3.73×10^{-2}	5	-	-	-	-	-	-
4JCV	42	3.73×10^{-2}	153	-	-	-	-	-	-	17	5.18×10^{-2}	3
3SZK	42	3.73×10^{-2}	40	32	7.20×10^{-2}	179	-	-	-	-	-	-
3S9D	42	3.73×10^{-2}	2	-	-	-	-	-	-	-	-	-
1RKE	42	3.73×10^{-2}	9	-	-	-	-	-	-	-	-	-
4HX3	42	3.73×10^{-2}	9	-	-	-	-	-	-	-	-	-
3HI6	-	-	-	-	-	-	-	-	-	17	5.18×10^{-2}	16
2GAF	42	3.73×10^{-2}	22	-	-	-	-	-	-	-	-	-
3BIW	21	2.68×10^{-3}	44	-	-	-	-	-	-	-	-	-
3RVW	-	-	-	32	7.20×10^{-2}	53	-	-	-	-	-	-
3AAA	21	2.68×10^{-3}	55	-	-	-	-	-	-	-	-	-
3R9A	42	3.73×10^{-2}	77	-	-	-	-	-	-	-	-	-
BP57	-	-	-	32	7.20×10^{-2}	88	-	-	-	-	-	-
4M76	-	-	-	32	7.20×10^{-2}	190	-	-	-	-	-	-
4GXU	42	3.73×10^{-2}	224	-	-	-	-	-	-	-	-	-

Table 5: This table shows the components of the R-SVM weight vectors in each feature direction, averaged over all the top 50 models, according to their model selection score, and over all c values, for the BM4 validation. A positive component indicates that, when comparing two decoys, the decoy with the more positive value for that feature is more likely to be a near-native. Conversely, a negative component implies that more negative values are associated with near-native structures. As the features are normalised as z-scores, the relative magnitude of the components relates to the relative importance of the features in the model. However, as the cumulative effects of all features are used for ranking, caution must be taken in interpreting these values in isolation. The features used are not orthogonal to one another, so the components of two highly discriminative features may be small but act in synergy, and thus a small component does not imply unimportance. Further, a large positive component may be offset by a positive component of an anticorrelated feature, or a negative component of a correlated feature. In addition, as this is a statistical learning method, a correlation between a feature and CAPRI categories does not imply causation. Subsequently, it would be incorrect to observe the component of ROT_S, the change in rotational entropy upon binding, and conclude that it is the most important factor for determining binding geometry, and perhaps more appropriate to speculate that it is a term that cannot be accounted for by the other features.

Feature	SwarmDock	pyDock	ZDOCK	SDOCK
ROT_S	-0.587	-0.585	-0.443	-0.273
CP_D1	-0.354	-0.389	-0.477	-0.312
CP_PIE	0.439	0.160	0.244	0.379
AP_calRW	0.739	-0.118	0.144	0.414
AP_calRWp	0.529	0.341	0.329	-0.242
ZRANK	-0.157	-0.233	-0.354	-0.154
CP_SJKG	-0.167	-0.059	-0.266	-0.380
AP_ACE	0.394	0.217	0.300	-0.106
CG_ENV	-0.172	-0.288	-0.062	-0.242
AP_dDFIRE	-0.111	-0.045	-0.319	-0.199
CG_PP	-0.030	-0.128	-0.238	-0.209
AP_OPUS_PSP	-0.082	-0.002	-0.149	-0.319
AP_DOPE	-0.046	-0.162	-0.189	-0.133
CP_Qp	-0.128	-0.026	-0.109	-0.252
CP_SKOIP	0.097	0.135	0.243	0.029
DDG_V	-0.133	-0.097	-0.100	-0.112
LK_SOLV	-0.441	-0.183	0.028	0.159
AP_PISA	-0.123	-0.068	-0.097	-0.148
CP_TS	0.026	0.179	0.099	0.107
AP_MPS	-0.043	-0.251	0.019	-0.128
AP_GOAP_DF	-0.039	0.005	-0.205	-0.163
ZRANK2	-0.187	0.090	0.340	0.141
CP_Qm	0.207	-0.058	0.121	0.108
CP_MJPL	0.084	0.072	-0.054	0.266
AA_PROP	-0.185	0.009	-0.200	0.029
AP_DOPE_HR	0.030	0.047	0.222	0.040
CP_MJ3h	-0.294	0.019	0.039	-0.102
Clust_0.45	-0.178	-0.021	-0.268	0.137
AP_T1	-0.119	-0.005	-0.103	-0.095
INSIDE	-0.046	-0.019	-0.070	-0.182
CG_BETA	-0.020	0.133	0.102	0.099
CP_TSC	-0.121	-0.059	-0.052	-0.077
CP_GKS	0.035	-0.067	-0.183	-0.088
CP_MJ2	-0.009	0.110	0.035	0.164
CP_HLPL	-0.147	0.054	0.260	0.117
CP_SKOb	0.103	0.104	-0.002	0.056
CP_BL	-0.080	-0.053	-0.121	-0.006
Clust_0.65	0.103	-0.084	-0.072	0.301
CP_TD	-0.019	-0.052	-0.072	-0.101
CP_MJ2h	0.296	-0.288	-0.182	-0.057
FA_ATR	-0.318	0.010	0.009	0.081
AP_DCOMPLEX	0.193	-0.004	-0.001	0.026
Clust_0.7	0.234	0.160	0.028	-0.218
CP_SKOa	-0.065	-0.087	0.200	0.149
Clust_0.35	0.155	0.053	0.159	-0.176
CP_RMFCEN1	0.013	-0.084	-0.079	-0.037
CP_BFKV	0.101	0.071	-0.046	0.049
Clust_0.4	0.173	-0.013	-0.024	0.022
AP_GEOMETRIC	-0.006	0.010	-0.116	-0.042
CP_MJ1	0.046	-0.133	-0.105	0.039
CP_TEs	0.043	0.019	0.017	0.069
AP_DDG_W	0.161	0.052	-0.023	-0.050

Feature	SwarmDock	pyDock	ZDOCK	SDOCK
AP_GOAP_ALL	-0.029	0.027	-0.097	-0.040
CP_Qa	0.007	0.072	-0.080	0.133
CP_BT	0.073	0.002	0.020	0.034
CP_RMFCEN2	-0.104	-0.059	0.009	0.027
HBOND	0.039	-0.108	-0.017	-0.031
FIREDOCK	0.101	0.282	-0.182	-0.089
AP_URS	0.097	-0.048	0.098	-0.037
NHB	0.096	-0.007	-0.004	0.020
FA_PP	0.077	-0.032	0.012	0.044
ROSETTADOCK	-0.049	0.074	-0.018	0.094
ROSETTA	-0.109	0.007	-0.005	0.010
Clust_0.6	-0.269	0.113	0.109	-0.041
FA_REP	0.071	0.009	-0.006	0.009
Clust_0.5	0.084	-0.079	0.092	-0.014
Clust_0.3	-0.147	-0.047	-0.016	0.130
CP_RO	-0.021	0.033	0.063	-0.004
CP_VD	-0.006	0.081	-0.029	0.019
PI_PI	-0.002	0.092	0.007	-0.032
CP_MSBM	-0.007	0.014	0.029	0.024
CP_TEl	0.038	-0.007	-0.013	0.038
AP_T2	0.056	-0.060	-0.058	0.005
AP_DARS	0.024	-0.017	-0.205	0.251
CP_MS	0.131	-0.129	0.104	-0.056
AP_DDG_U	-0.046	0.018	0.007	0.073
CAT_PI	0.050	0.004	-0.019	-0.085
CP_DDG_U	0.014	0.073	-0.013	-0.025
CG_VDW	0.003	0.081	-0.087	0.048
Clust_0.55	0.074	-0.036	0.000	-0.081
FIREDOCK_EI	-0.124	-0.216	0.062	0.320
FIREDOCK_AB	-0.064	-0.050	0.030	0.044
AP_W1	-0.007	-0.082	0.065	-0.005
AP_DFIRE2	-0.334	-0.218	0.342	0.191
CP_DDG_W	0.001	-0.028	0.016	0.030
CP_RMFCA	-0.017	-0.069	0.005	0.067
HBOND2	0.060	0.043	-0.036	-0.054
ALIPH	0.053	-0.004	-0.082	0.043
AP_GOAP_G	-0.013	0.021	-0.027	0.010
CP_TB	0.045	-0.067	0.004	0.026
TRANS_S	0.000	0.000	0.000	0.000

References

Andrusier, N., Nussinov, R. & Wolfson, H. J. (2007) FireDock: fast interaction refinement in molecular docking. *Proteins,* **69** (1), 139–159.

Brooks, B. R., Brooks, C. L., Mackerell, A. D., Nilsson, L., Petrella, R. J., Roux, B., Won, Y., Archontis, G., Bartels, C., Boresch, S., Caflisch, A., Caves, L., Cui, Q., Dinner, A. R., Feig, M., Fischer, S., Gao, J., Hodoscek, M., Im, W., Kuczera, K., Lazaridis, T., Ma, J., Ovchinnikov, V., Paci, E., Pastor, R. W., Post, C. B., Pu, J. Z., Schaefer, M., Tidor, B., Venable, R. M., Woodcock, H. L., Wu, X., Yang, W., York, D. M. & Karplus, M. (2009) CHARMM: the biomolecular simulation program. *J Comput Chem,* **30** (10), 1545–1614.

Chaudhury, S., Lyskov, S. & Gray, J. J. (2010) PyRosetta: a script-based interface for implementing molecular modeling algorithms using Rosetta. *Bioinformatics,* **26** (5), 689–691.

Chowdhury, R., Rasheed, M., Keidel, D., Moussalem, M., Olson, A., Sanner, M. & Bajaj, C. (2013) Protein-protein docking with F(2)Dock 2.0 and GB-rerank. *PLoS ONE,* **8** (3), e51307.

Chuang, G. Y., Kozakov, D., Brenke, R., Comeau, S. R. & Vajda, S. (2008) DARS (Decoys As the Reference State) potentials for protein-protein docking. *Biophys. J.,* **95** (9), 4217–4227.

Feng, Y., Kloczkowski, A. & Jernigan, R. L. (2010) Potentials 'R' Us web-server for protein energy estimations with coarse-grained knowledge-based potentials. *BMC Bioinformatics,* **11**, 92.

Huang, S. Y. (2015) Exploring the potential of global protein-protein docking: an overview and critical assessment of current programs for automatic ab initio docking. *Drug Discov. Today,* **20** (8), 969–977.

Liu, S. & Vakser, I. A. (2011) DECK: Distance and environment-dependent, coarse-grained, knowledge-based potentials for protein-protein docking. *BMC Bioinformatics,* **12**, 280.

Liu, S., Zhang, C., Zhou, H. & Zhou, Y. (2004) A physical reference state unifies the structure-derived potential of mean force for protein folding and binding. *Proteins,* **56** (1), 93–101.

Lu, H., Lu, L. & Skolnick, J. (2003) Development of unified statistical potentials describing protein-protein interactions. *Biophys. J.,* **84** (3), 1895–1901.

Lu, M., Dousis, A. D. & Ma, J. (2008) OPUS-PSP: an orientation-dependent statistical all-atom potential derived from side-chain packing. *J. Mol. Biol.,* **376** (1), 288–301.

Mintseris, J., Pierce, B., Wiehe, K., Anderson, R., Chen, R. & Weng, Z. (2007) Integrating statistical pair potentials into protein complex prediction. *Proteins,* **69** (3), 511–520.

Moal, I. H., Dapkunas, J. & Fernandez-Recio, J. (2015) Inferring the microscopic surface energy of protein-protein interfaces from mutation data. *Proteins,* **83** (4), 640–650.

Moal, I. H. & Fernandez-Recio, J. (2013) Intermolecular Contact Potentials for Protein-Protein Interactions Extracted from Binding Free Energy Changes upon Mutation. *J Chem Theory Comput,* **9** (8), 3715–3727.

Moal, I. H., Jiménez-García, B. & Fernández-Recio, J. (2015) CCharPPI web server: computational characterization of protein-protein interactions from structure. *Bioinformatics,* **31** (1), 123–125.

Ohue, M., Matsuzaki, Y., Uchikoga, N., Ishida, T. & Akiyama, Y. (2014) MEGADOCK: an all-to-all protein-protein interaction prediction system using tertiary structure data. *Protein Pept. Lett.,* **21** (8), 766–778.

Pierce, B. & Weng, Z. (2007) ZRANK: reranking protein docking predictions with an optimized energy function. *Proteins,* **67** (4), 1078–1086.

Pierce, B. & Weng, Z. (2008) A combination of rescoring and refinement significantly improves protein docking performance. *Proteins,* **72** (1), 270–279.

Rajgaria, R., McAllister, S. R. & Floudas, C. A. (2006) A novel high resolution Calpha–Calpha distance dependent force field based on a high quality decoy set. *Proteins,* **65** (3), 726–741.

Rajgaria, R., McAllister, S. R. & Floudas, C. A. (2008) Distance dependent centroid to centroid force fields using high resolution decoys. *Proteins,* **70** (3), 950–970.

Ravikant, D. V. & Elber, R. (2010) PIE-efficient filters and coarse grained potentials for unbound protein-protein docking. *Proteins,* **78** (2), 400–419.

Schneidman-Duhovny, D., Rossi, A., Avila-Sakar, A., Kim, S. J., Velazquez-Muriel, J., Strop, P., Liang, H., Krukenberg, K. A., Liao, M., Kim, H. M., Sobhanifar, S., Dotsch, V., Rajpal, A., Pons, J., Agard, D. A., Cheng, Y. & Sali, A. (2012) A method for integrative structure determination of protein-protein complexes. *Bioinformatics,* **28** (24), 3282–3289.

Shen, M. Y. & Sali, A. (2006) Statistical potential for assessment and prediction of protein structures. *Protein Sci.,* **15** (11), 2507–2524.

Tobi, D. (2010) Designing coarse grained-and atom based-potentials for protein-protein docking. *BMC Struct. Biol.,* **10**, 40.

Tobi, D. & Bahar, I. (2006) Optimal design of protein docking potentials: efficiency and limitations. *Proteins,* **62** (4), 970–981.

Torchala, M., Moal, I. H., Chaleil, R. A., Fernández-Recio, J. & Bates, P. A. (2013) SwarmDock: a server for flexible protein-protein docking. *Bioinformatics,* **29** (6), 807–809.

Viswanath, S., Ravikant, D. V. & Elber, R. (2013) Improving ranking of models for protein complexes with side chain modeling and atomic potentials. *Proteins,* **81** (4), 592–606.

Vreven, T., Moal, I. H., Vangone, A., Pierce, B. G., Kastritis, P. L., Torchala, M., Chaleil, R., Jiménez-García, B., Bates, P. A., Fernández-Recio, J., Bonvin, A. M. & Weng, Z. (2015) Updates to the Integrated Protein-Protein Interaction Benchmarks: Docking Benchmark Version 5 and Affinity Benchmark Version 2. *J. Mol. Biol.,* **427** (19), 3031–3041.

Yang, Y. & Zhou, Y. (2008*a*) Specific interactions for ab initio folding of protein terminal regions with secondary structures. *Proteins,* **72** (2), 793–803.

Yang, Y. & Zhou, Y. (2008*b*) Ab initio folding of terminal segments with secondary structures reveals the fine difference between two closely related all-atom statistical energy functions. *Protein Sci.,* **17** (7), 1212–1219.

Zhang, J. & Zhang, Y. (2010) A novel side-chain orientation dependent potential derived from random-walk reference state for protein fold selection and structure prediction. *PLoS ONE,* **5** (10), e15386.

Zhou, H. & Skolnick, J. (2011) GOAP: a generalized orientation-dependent, all-atom statistical potential for protein structure prediction. *Biophys. J.,* **101** (8), 2043–2052.
